# Clays as Inhibitors of Polyurethane Foams’ Flammability

**DOI:** 10.3390/ma14174826

**Published:** 2021-08-25

**Authors:** Aleksander Hejna

**Affiliations:** Department of Polymer Technology, Gdańsk University of Technology, Narutowicza 11/12, 80-233 Gdańsk, Poland; aleksander.hejna@pg.edu.pl or ohejna12@gmail.com

**Keywords:** polyurethanes, polyurethane foams, layered silicates, thermal stability, flammability, clays

## Abstract

Polyurethanes are a very important group of polymers with an extensive range of applications in different branches of industry. In the form of foams, they are mainly used in bedding, furniture, building, construction, and automotive sectors. Due to human safety reasons, these applications require an appropriate level of flame retardance, often required by various law regulations. Nevertheless, without the proper modifications, polyurethane foams are easily ignitable, highly flammable, and generate an enormous amount of smoke during combustion. Therefore, proper modifications or additives should be introduced to reduce their flammability. Except for the most popular phosphorus-, halogen-, or nitrogen-containing flame retardants, promising results were noted for the application of clays. Due to their small particle size and flake-like shape, they induce a “labyrinth effect” inside the foam, resulting in the delay of decomposition onset, reduction of smoke generation, and inhibition of heat, gas, and mass transfer. Moreover, clays can be easily modified with different organic compounds or used along with conventional flame retardants. Such an approach may often result in the synergy effect, which provides the exceptional reduction of foams’ flammability. This paper summarizes the literature reports related to the applications of clays in the reduction of polyurethane foams’ flammability, either by their incorporation as a nanofiller or by preparation of coatings.

## 1. Introduction

Polyurethanes (PUR) are used in a wide range of industries due to their adjustable parameters [1]. Nearly 50% of the total polyurethane market consists of CASE (coatings, adhesives, sealants, and elastomers), while the other half includes rigid and flexible forms. The application requirements of polyurethane foams are determined by their mechanical properties (tensile or compressive strength, brittleness, hardness, and resilience), physicochemical properties (density, thermal conductivity coefficient), and flammability [2,3].

Flexible polyurethane foams have gained a significant position among foam materials due to their application in the furniture industry, mainly in mattresses production, where their light weight and mechanical performance can be combined. It is associated with the open cell structure leading to increased flexibility, but also high water and air permeability [4]. They are also used for packaging production, where they serve as anti-shock material that protects products against mechanical damages [5]. Their advantage is the ease of manufacturing products with very complicated shapes, using cheap and straightforward molds, or foaming directly around the packed item.

On the other hand, rigid foams are characterized by high content of closed cells, which, along with their low apparent density, stiffness, mechanical strength, low water permeability, good chemical resistance, and, above all, excellent thermal insulating properties, is determining their applications [6,7]. The main ones are thermal insulation and structural materials for refrigeration equipment and construction [8,9]. The use of rigid polyurethane foams as thermal insulating materials in construction is an important issue due to energy savings [10]. It is estimated that globally about 40% of the energy consumed is used to maintain proper temperature in buildings, both through heating and air conditioning. This value gives an idea of how vital good thermal insulation in buildings is from the point of view of energy consumption, which is currently often emphasized considering the Passive Housing concept [11,12]. An important feature of these materials is the possibility to apply them on site as sprayed foams, which enables the manufacturing of thermal insulation without joints, which significantly reduces heat loss due to gaps or seams. This translates into energy efficiency and reduced costs of heating/air conditioning of buildings. This product can also be used in hard-to-reach places without cutting. The use of rigid polyurethane foam guarantees additional stiffening of the roof structure, which may increase the life span of buildings. There is no “dusting phenomenon” during the application of the insulation foam, which makes it safe for health. It constitutes a tight barrier for dust and pollen. Appropriate application of foam on metal elements increases anti-corrosion properties [13].

Irrespective of the type of polyurethane foams, among the requirements for these materials, except for the mechanical and insulation performance, crucial is their flammability. Devoid of flame retardants, PUR foams are flammable materials releasing significant amounts of toxic gases and fumes during combustion [14]. Therefore, foams should be properly modified to meet the criteria resulting from legal regulations for furniture, construction, and insulation materials [15]. Considering furniture, the most important are US Consumer Product Safety Commission Standard for the Flammability of Upholstered Furniture [16] and Furniture and Furnishings (Fire) (Safety) Regulations of the Furniture Industry Research Association [17]. In the case of insulation materials, regulations are very location-dependent, but in the European Union (EU) the main documents are the Commission Decision of 9 September 1994 implementing Article 20 of Directive 89/106/EEC on construction products and Commission Decision of 8 February 2000 implementing Council Directive 89/106/EEC as regards the classification of the reaction to fire performance of construction products [18,19], which were the basis for implementation of the EUROCLASS system. Moreover, various international regulations, like the Stockholm Convention [20] or the Basel Convention [21], banned the use and trade of various chemicals, including multiple flame retardants, which were found most harmful towards human health and the environment.

Having in mind current pro-ecological trends, the law regulations dealing with the flammability of materials are more and more demanding, considering both performance of materials, as well as the incorporated chemicals. Therefore, multiple research works focus on the development and evaluation of environmentally friendly flame retardants often based on natural organic and mineral materials, such as clays [22,23,24]. There are several comprehensive review papers dealing with the flammability of polyurethanes [25,26,27,28]. However, they are not always up-to-date and mostly they often hardly touched on the application of clays as flame retardants. The aim of the presented review work was systematic summary of research addressing flammability of foamed polyurethane/clay composites in order to provide in-depth information for scientists working in the field and, possibly, the industrial environment. Different state-of-the-art strategies applied in the improvement of flame retardancy of PUR foams using clays are reviewed and discussed.

## 2. Polyurethanes—Production and Market Size

**Figure 1:**
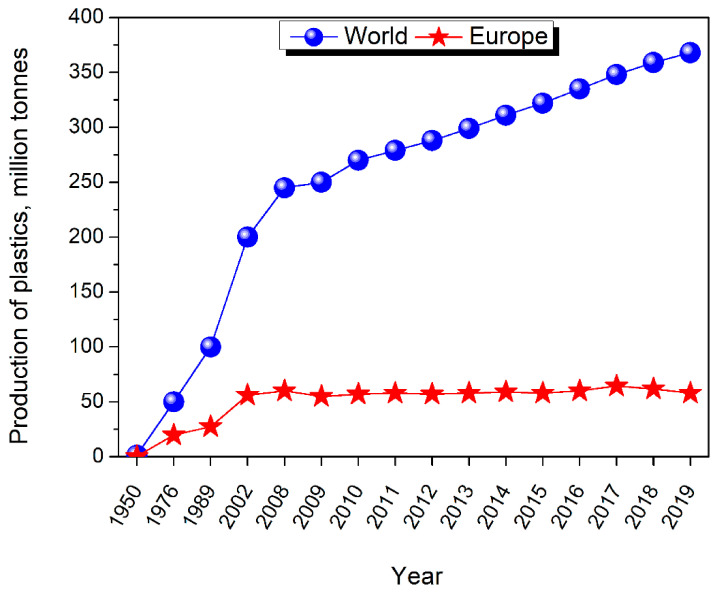
The global and European production of plastics over the last decades.

The plastics industry is a very dynamically developing branch of the chemical industry due to the multiplicity of applications and the possibility of selecting a suitable material with properties desired for almost any application. Over the last decades, there has been a significant increase in the global production of plastics, as shown in Figure 1 [29,30,31]. The presented statistics do not include the production of various types of fibers obtained from polymeric materials, such as poly(ethylene terephthalate) (PET), polypropylene (PP), polyamide (PA), or polyacrylic fibers [32].

The dynamic growth of the European market was somewhat hampered by the global economic crisis and the fact that some companies moved their production to Asian countries due to lower production costs. Therefore, the European share in global plastics production decreased from 23 to 20% between 2007 and 2015 [33]. According to the reports of PlasticsEurope, the biggest consumer of plastics is the packaging market, which consumes almost 40% of the total plastic production [34]. Around 20% of European plastics production goes to the construction industry. Polyethylene, polypropylene, and polyethylene terephthalate dominate the packaging market. On the engineering plastics market, polyvinyl chloride, high-density polyethylene, expanded polystyrene, and polyurethanes are the most popular [34].

For polyurethanes, Asian countries are by far the largest producer. In 2012, Asian PUR production was about 10 million tons, reaching 13.1 million tons in 2021 [35]. At the same time, Europe produced about 4.8 million tons in 2012 and 6.5 million tons in 2017 [36]. It is projected to reach 7.5 million tons in 2022, which is expected to be associated with strong production growth in Eastern Europe [37]. Thus, it can be seen that economic growth in many regions of the world is causing both production and demand for PUR to increase steadily. In China, the driving force for the polyurethane industry is the significant increase in demand for materials used in construction [38]. In other regions of the world, the demand for PUR materials is equally high, with polyurethanes ranking fifth in Europe in terms of specific plastics demand (7.5%) [39]. Generally, the current global demand for polyurethanes is estimated at 20.4 million tons [40] and is expected to grow by 10% in the next three years [41]. Therefore, all of the works dealing with the polyurethane materials are of high-value, because their results could affect a lot of people all over the world.

## 3. Polyurethanes—Flammability

### 3.1. General Information

The flammability of polymer materials determines the material’s susceptibility to flame combustion, glowing, smoldering, or smoking. Combustion is a comprehensive concept and is defined as a physico-chemical phenomenon during which the polymer is thermally destructed, gaseous mixture ignited, flames propagated, heat and radiation emitted, toxic gaseous substances released, and self-extinguished [42,43]. Statistical data and studies on fires indicate that in recent years the vast majority (60–80%) of fatalities are caused by inhalation of toxic products of thermal decomposition and combustion [44]. The source of these substances is burning upholstery, flooring, and bedding materials. Their presence also reduces visibility during a fire.

The stages of polymer material combustion can be presented in the following sequence: initiation of combustion called ignition, followed by the phase of fire ignition and accompanying fire flare, then the stage of intensive combustion, i.e., the vigorous development of flames, and the whole process ends with fire extinguishing [45].

The phenomenon of polymer combustion depends on many factors. This process is significantly influenced by the composition, chemical structure, and density [46]. In addition, the rate of fire spread is determined by the surface properties of the material. Decisive here is the roughness of the external surface, the porosity of the surface of the internal structure, shape, and volume of the product, and the way it is distributed in the room. The combustion phenomenon also depends on the concentration profile of the substrates and products at each stage of combustion and the heat released, and the method of its dissipation. The ignition conditions or the initiation of combustion, the time taken to heat the material, and the energy required for ignition must also be considered. The fate of the combustion may be determined by material properties, such as combustion heat, specific heat, and thermal conductivity.

The classification of flames, based on the phase state of the reactants, specifies the following types of combustion: homogeneous, heterogeneous, and homogeneous–heterogeneous (mixed) combustion [47]. Homogeneous combustion is characterized by the lack of separation between reactants and occurs in the gas phase above the surface of the combustible material in the form of a flame. Evaporation, sublimation, or pyrolysis results in the release of large amounts of volatile combustible products. On the other hand, heterogeneous combustion occurs at the phase boundary and is located on the surface of the combustible solid. The phenomenon is accompanied by smoldering, resulting in the formation of embers and the appearance of light, but without a visible flame [48]. The most common type of polymer combustion is mixed combustion, containing both homogeneous and heterogeneous combustion.

The mechanism of flame formation is defined by two characteristic chemical reactions: a global (self-accelerating) exothermic momentum chemical reaction; and a local momentum exothermic fuel oxidation reaction [49,50]. The former leads to ignition of the measurement material, as a consequence of which an explosion may occur. Ignition occurs by spontaneous combustion, which is a spontaneous phenomenon. The exothermic momentum of the oxidation reaction occurs locally and is initiated by an external influence at a specific location of the material [51]. In contrast to spontaneous ignition, this reaction involves a more complex process—forced ignition. In the case of forced ignition, the intervention of an external agent is unavoidable, which can be, among others, a significantly elevated temperature, a cigarette butt, another flame, an electric spark, or intense radiation [27].

As mentioned above, polyurethane foams function among plastics as materials with versatile properties and a wide range of industrial applications [52]. Nowadays, more and more attention is being paid to improving the flammability properties of polyurethanes, which in turn determine their use in automotive applications, mattresses, and insulation boards in construction [53,54]. Environmental factors also drive such an approach to reduce plastics’ environmental impacts [55].

**Figure 2:**
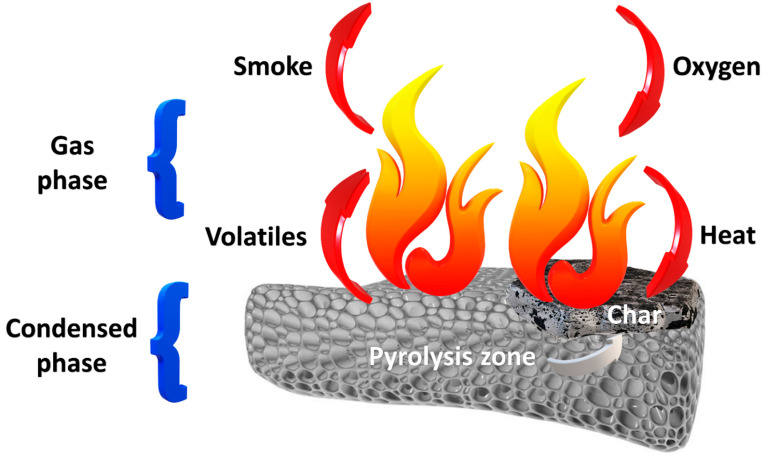
The general scheme of polyurethane foams’ combustion.

Conventional polyurethane foams are flammable materials. They ignite from a small fire source and burn at high rates. Combustion is accompanied by the release of heat and the formation of smoke and toxic gases, which is presented in Figure 2 [56]. Decomposition of polyurethane material occurs at temperatures above 200 °C. The products of polyurethane decomposition are mainly hydrogen cyanide and carbon monoxide, but nitric oxides, nitriles, hydrogen chloride, and carbon dioxide are also found [57]. The simplest compounds are generated at temperatures exceeding 800 °C, due to defragmentation of previous decomposition products [58]. Table 1 presents the most common combustion products of polyurethanes. Vapors of isocyanates float above the surface of the burnt polymer and condense, while liquid polyols undergo further decomposition.

The pyrolysis process (temp. 80–150 °C) breaks hydrogen bonds between oxygen and hydrogen atoms in urethane groups [59]. Hydrogen bonds between other chemical groups also break in the 130–200 °C range. The urethane bonds are characterized by increased resistance to oxygen at elevated temperatures [60]. Less stable groups in the chain and thermal decomposition products of the urethane group undergo oxidation reactions. The type and composition of pyrolysis products depend on the chemical structure of the polyurethane. In the polymer obtained from MDI, the main volatile products of pyrolysis are phenyl isocyanate, p-toluyleneisocyanate (TDI), o-benzodinitrile, isoquinoline, and hydrocarbons, such as benzene, toluene, xylene, and naphthalene [61]. TDI-based polyurethane, at temperatures above 200 °C, decomposes with the release of acetonitrile, acrylonitrile, benzonitrile, aniline, pyridine, and pyrrole [62]. Among the non-volatile decomposition products, benzonitrile predominates, which undergoes further decomposition and leads to HCN [62].

The type of polyurethane foam material, the degree of crosslinking, and the physical state of the product affect flammability [60]. The highly developed pore surface of foams, open-cell structure, low thermal conductivity, and low density facilitate combustion. The ignition conditions determine the susceptibility of the foam to ignition. Polymer combustion, otherwise known as a multi-step process, is usually initiated by forced ignition. The initiation of the oxidation reaction occurs after the initial heating of the material, which is local and superficial and leads to the combustion focus. According to the definition, the cause of ignition of the gaseous mixture is thermo-oxidative decomposition, within which depolymerization and destruction of the polymer occur. The formation of the fire depends on the diffusion of the reactants. In the case of polyurethane foam, oxygen can easily pass through the cells of the combustible material. Mixing of the combustible reactants and the oxidant is followed by pyrolysis, which the supply of energy can locally induce. The energy can result from a mechanical or electrical impulse or the touch of a glowing flame, among other things. Ignition is evidenced by the appearance of smoldering or glowing and an increase in temperature. Flexible polyurethane foams have lower melting and ignition temperatures than rigid foams [60].

### 3.2. Flame Retardancy of Polyurethanes

The inhibition of flammability of foamed polymeric materials while maintaining or improving mechanical properties is achieved by flame-retardant modification through the addition of flame-retardant compounds, called flame retardants. The application of flame retardants can also positively affect other material characteristics, such as thermo-stability, reduction of thermal destruction rate, toxic combustion products, and slow down the coke formation process. The content of catalysts in the reaction mixture (polyols and isocyanates) can change the mechanism leading to macromolecules that contain different amounts of other groups besides the urethane group. Thus, modification refers to changing the chemical structure of macromolecules, resulting in a material with desired properties. Usually, even a tiny change in the chemical structure can significantly change the properties of the final product.

The selection of an appropriate flame retardant for plastic is based on the addition of a substance that meets specific requirements. The desired characteristic effectively prevents fires, resulting in the expected absence of emission of toxic substances during combustion and the absence of smoke [28]. The applied combustion inhibitor must be compatible with the polymer. Thus it cannot adversely affect the processing, mechanical, and aging properties. Under thermooxidative conditions, the temperature of thermal decomposition of the flame retardant should be lower than that of the polymer. The flame retardants should also be resistant to elevated temperatures, UV radiation, and water. Advantageously, they prevent the dripping of the melted polymer composite. The economic aspect is also a key factor for the application of a specific flame retardant.

General methods to reduce the flammability of polyurethane foams include diluting the reaction mixture with non-flammable agents. The addition of flame retardants, which show chemical activity in combustion processes, can be done at the stage of macromolecular compounds processing (additive flame retardants) or reaction with foam components at the stage of their synthesis (reactive flame retardants) [63]. The solution can be the use of flame-retardant aromatic polyisocyanates during synthesis. Increasing the degree of crosslinking of the polymer and increasing the relative content of aromatic rings are also helpful. It is advantageous to obtain heat-resistant chemical bonds at the foaming stage.

#### 3.2.1. Mechanisms of Action of Flame Retardants

Depending on their nature and potential interactions with the particular polymer matrix, flame retardants are divided into reactive and additive ones. Reactive (reacting with the polymer) flame retardants are introduced into plastics to chemically modify them by incorporating atoms of flame-retardant elements into the macromolecular structure. Their advantage is the permanent bonding of the flame retardant to the polymer, resulting in significantly reduced volatilization during use [70]. The chemical bonds also prevent migration to the surface of the plastic [71]. They do not function as a plasticizer and are not deteriorating the thermal stability of the composite. Flame retardants such as tetrabromodiene, pentabromophenol, or hexabromophthalate are mainly used for thermosetting plastics (polyester resins, epoxy resins, polyurethanes, etc.). They can be introduced into the polymer chain during polymerization.

Additive flame retardants are incorporated at the processing stage of macromolecular compounds. In their case, there is no chemical bonding with atoms of the polymer’s molecular structure. They can additionally serve as plasticizers or fillers [72]. Their compatibility with the polymer is required, enabling the homogenous distribution in the polymer matrix and resulting in the consistent performance of the material.

The effect of flame retardants on the combustion of polymeric materials is a very complex process, which can co-occur according to several mechanisms, involving numerous chemical reactions and physical interactions [73]. Polymer ignition by diffusion occurs by mixing the oxidant with flammable reactants, i.e., volatile thermal decomposition products. Therefore, the thermal stability of polyurethane plays a vital role in flame retardancy effectiveness [25]. The flame retardant is expected to be activated as early as the thermal destruction stage of the polymer. The action of flame retardants follows two mechanisms: chemical and physical. The common feature of both is reducing the rate of formation of volatile, flammable, pyrolysis products.

##### Chemical Mechanism

**Figure 3:**
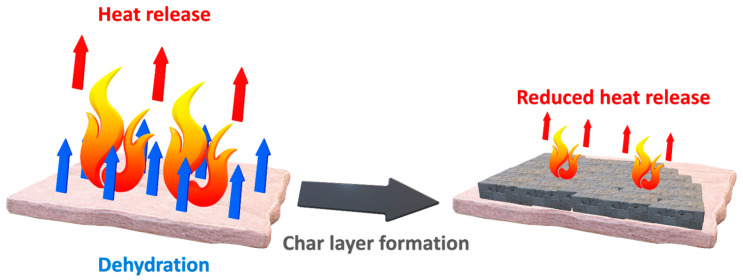
Scheme of char layer formation during dehydration of flame retardants.

The chemical mechanisms of flame retardants can be divided into two main groups, reactions in condensed phase and in gas phase. The first group is mostly based on the generation of char layer on the surface of burning polymer, which could be the result of flame retardant dehydration, cross-linking or other reactions, as presented in Figure 3. Such reactions may significantly increase the viscosity of molten polymer on the surface of burning material, which reduces the heat and mass transfer inhibiting the flame. Moreover, formation of char layer often promotes the stabilization of polymer structure and protects the insight of burning material.

Considering the gas phase, the chemical flame retardancy is mostly based on free radical scavenging and interrupting the combustion. The products of flame retardants’ decomposition are combining with highly reactive H and OH radicals, which are very important in fire propagation. As a result, the inactive molecules or significantly less reactive radicals are generated, so the combustion is inhibited. Such mechanism is mostly emphasized when halogen flame retardants are applied [74].

##### Physical Mechanism

**Figure 4:**
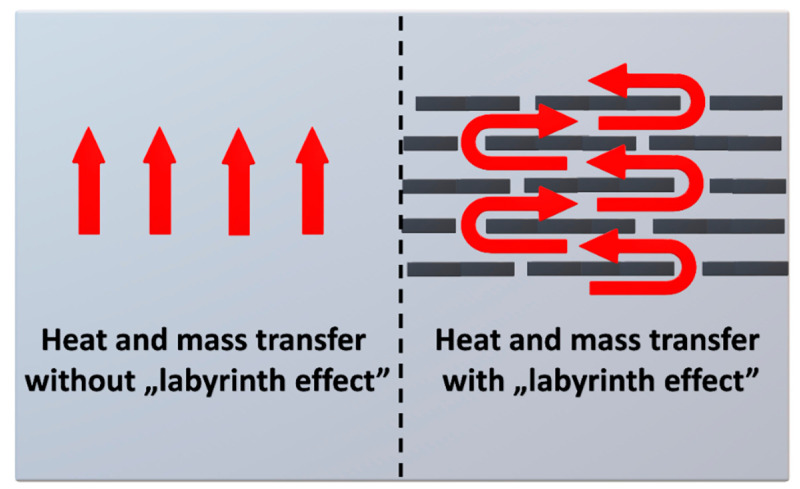
The impact of “labyrinth effect” on the heat and mass transfer inside the material.

The physical flame retardants improve the performance of polymers due to a series of phenomena. Flame retardants affect the change in the thermal state of the material. Delaying the pyrolysis process and decreasing the temperature rise on the material surface is achieved by additives with high thermal conductivity, distributing the heat flowing from the flame throughout the material volume [75]. Moreover, proper additives characterized by moderate decomposition temperature may break down endothermically and remove the heat from the combustion system. Another method is reducing the degree of the macromolecular compound thermo-oxidative decomposition, which occurs due to reducing the amplitude of thermal fluctuations of its macromolecules. By creating barrier layers, flame inhibitors retard or block the energy and mass transfer between the combustion zones—solid and gaseous [76]. A physical barrier layer is often created by layered clays, due to their flake-like shape, resulting in generation of so-called “labyrinth effect”, presented in Figure 4. Flame retardants may significantly influence the release of non-flammable gases, such as CO_2_, H_2_O, NH_3_, and SO_2,_ as a result of their thermal decomposition, simultaneously diluting the combustible gases. Poor combustion mixture, created due to diluting volatile pyrolysis products with non-flammable gases, and containing lower concentration of oxygen, is incapable of spontaneous ignition or sustained combustion [77]. Flame retardants contribute to prolonging the onset time of pyrolysis. Examples of flame retardants exhibiting physical effects are aluminum hydroxide Al(OH)_3_ and magnesium hydroxide Mg(OH)_2_.

### 3.3. Classification of Flame Retardants for Polyurethanes

The variety of structures and the mode of action of flame retardants on plastics means no universal additive, which is compatible with every polymer. Commonly used flame retardants contain chlorine, bromine, phosphorus, nitrogen, aluminum, boron, and antimony [78]. The most popular group of additives are halogen compounds. The flame-retardant action concerns the gas phase and condensed phase, as a result of synergic systems, both phases [79]. However, during the combustion of materials with halogen flame retardants, toxic gases (hydrogen chloride, bromine) are released—corrosive, irritating, toxic, and causing smoke. As a result, restrictions have been placed on their use in European Union member states, increasing research on other groups of flame retardants [80].

An alternative to the use of halogen compounds is the addition of non-toxic inorganic compounds. Flame retardation of polymeric material is realized by introducing additive compounds that do not bond with the polymer. Currently, the aim is to use halogen-free flame retardants, whose introduction in a relatively small amount would produce the desired properties. For this purpose, the most common are phosphorus and nitrogen compounds, metal hydroxides, expanded graphite, and nanofillers.

#### 3.3.1. Nitrogen-Based Flame Retardants

One of the most popular groups of flame retardants used for polyurethanes are nitrogen-based compounds and, among them, the most essential is melamine and its derivatives [81]. The high efficiency of melamine in reducing the flammability of polymer materials is attributed to the complex mode of action. It absorbs the heat and generates non-combustible nitrogen-based gases resulting from thermal decomposition, mainly ammonia, which acts as diluent [82]. Melamine is considered to increase the head capacity of the material and combustion system, so its surface temperature is reduced, inhibiting the ignition. Above 250 °C, the melamine degrades, forming various oligomeric and polymeric products, such as melam, melem, or melon, whose stability exceeds melamine (~350, 450, and 600 °C, respectively) [83]. The final stage of decomposition and deamination is the formation of graphitic carbon nitride generating the protective layer for polyurethane. Generally, the melamine and its derivatives were extensively examined as flame retardants for polyurethane materials [84,85].

#### 3.3.2. Phosphorus-Based Flame Retardants

Commonly used flame retardants are organic and inorganic phosphorus compounds. This type of flame retardant’s wide range of applications is evidenced by the lack of release of toxic combustion products, reduced smoke emission, and increased flame retardancy [86]. The use of phosphorus flame retardants reduces the flammability of the polymer. Also, it contributes to forming a protective layer on the surface of the material, characterized by a low heat-transfer coefficient and low diffusion coefficient, which hinders further combustion [87]. Phosphorus compounds exhibit flame-retardant activity in the gas phase, condensed phase, and also in the case of both phases. They are considered the most effective combustion inhibitors in the gas phase due to their ability to bind radicals. In the solid phase, phosphorus-containing polymers undergo thermal decomposition with the release of phosphoric and polyphosphoric acids. The acids lead to the dehydrogenation of the polymer, resulting in the formation of a highly viscous liquid film on the surface of the combusted material, which limits the transport of mass and heat between the gas and condensed phases [88]. Organophosphorus compounds occupy a unique position among phosphorus flame retardants, which include, among others: organic phosphates (V); phosphates (III); diphosphates (V); pyrophosphates; and phosphorus halides. They are flame-retardant additives for thermoplastics and thermosets and can also act as plasticizers for polymers such as poly(vinyl chloride) (PVC) [89].

The use of compounds containing not only phosphorus but also nitrogen atoms in their structure is also widespread. Phosphorus–nitrogen synergism is a resultant effect in combustion suppression, caused by the presence of phosphorus and nitrogen compounds, such as urea (NH_2_CONH_2_) and melamine (C_3_N_3_(NH_2_)) or the use of poly(ammonium phosphates) [90]. The obtained effect is not dependent on the nitrogen–phosphorus weight ratio. Urea/phosphorus and melamine/phosphorus systems accelerate the formation of polyphosphoric acid, which can reduce combustion [91]. To exploit the synergism between nitrogen and phosphorus atoms, compounds such as aminophosphonates, now widely used as biologically active substances, are also introduced into polymers [92].

#### 3.3.3. Inorganic Flame Retardants

Aluminum hydroxide (ATH) and magnesium hydroxide (MDH) represent inorganic halogen-free flame retardants. The advantages of their use are low smoke generation and toxicity, negligible corrosivity, accessible storage, and, in comparison with halogen inhibitors, low cost [93]. The mechanism of action of halogen-free flame retardants of mineral origin proceeds by endothermic dehydration.

Endothermic decomposition with steam release occurs in contact with fire at 180 °C for ATH and 330 °C for MDH [94]. The steam dilutes the volatile products of thermal decomposition and reduces smoke emission. The oxides formed during dehydration create a protective layer on the material’s surface, thereby restricting the movement of oxygen and flammable gases into the plastic [95].

Aluminum hydroxide Al(OH)_3_, the most common flame retardant because of its relatively low decomposition temperature, has been used to flame retard polymers whose processing temperature does not exceed 200–220 °C [96]. ATH is used for elastomers, thermoplastics, and resins [97]. Magnesium hydroxide Mg(OH)_2_ is used mainly to reduce the flammability of thermoplastic and thermoset materials [98]. The use of hydroxides as flame retardants, besides numerous advantages, also has disadvantages. A disadvantage is a necessity of using the compounds in high concentrations (often over 50%), which significantly contributes to the deterioration of the mechanical performance [99].

#### 3.3.4. Expandable Graphite

Expandable graphite is an exciting material, which can be applied as flame retardants of various materials. It consists of graphite flakes intercalated using sulfuric acid or other strong oxidizers [100]. When subjected to the head source, such as fire, it expands even hundreds of times, creating a carbonaceous layer, which protects the material’s surface. The protective effect is based on the limited heat and mass transfer, which inhibits the fire spread [101]. Moreover, during combustion, both carbon and sulfur are oxidized, resulting in a non-flammable gas mixture of water vapor, sulfur, and carbon dioxides. Except for the dilution of the gas phase, the oxidation process consumes enormous amounts of oxygen from the atmosphere, naturally inhibiting the fire [102]. Because of these advantages, expandable graphite was extensively investigated as a flame retardant of polyurethane foams [103,104,105]. Nevertheless, to provide satisfactory results, it has to be applied in relatively high contents, sometimes even exceeding 20 wt%, which affects the processing and mechanical performance of foams [106]. Therefore, it can be effectively combined with the other types of flame retardants, mostly phosphorus-based, which allows reducing its loadings enhancing the mechanical aspects of material providing the flammability-oriented synergy effect [107,108].

#### 3.3.5. Clays

Nanofillers were initially intended to play a similar role as their microscale counterparts, i.e., primarily to improve the mechanical properties of composites. However, research has shown that the use of nanofillers in composites also brings benefits related to the flammability and fire behavior of composites. In the 1970s and 1980s, the first patents appeared, which referred to the benefits of reduced flammability of composites resulting from the use of aluminosilicate nanofillers [109]. At first, these were only reports of increased flame resistance that were not supported by any data, as in the case of the Toyota patent [110]. Bradbury et al. [111] used vermiculite, which caused increased charring of the composite and spontaneous flame extinction. Their competitors at DuPont, in their patents, also presented the use of aluminosilicates to flame retard composites. Still, in this case, the aluminosilicate nanoparticles were to act as drip-reducing compounds in composites flame-retarded with traditional flame retardants. Like in the other patent [111], increased charring of the composite during burning was also reported [112]. The next more significant reports were in the second half of the 1990s and the first decade of the 21st century. Work by Jeffrey Gilman of the National Institute of Standards and Technology in Gaithersburg showed that the presence of nanometer-sized montmorillonite (MMT) particles in a polymer composite significantly improved fire behavior [113,114]. Gilman and many other researchers studied various nanofillers, not only aluminosilicates but also substances such as titanium dioxide nanoparticles, carbon nanotubes, nanosilica, and silsesquioxanes [115,116,117,118,119]. All of these materials reduced the flammability of the composites compared to pure plastics while improving the physical, mechanical, and barrier properties of the composites, which was a considerable advantage of nanofillers over traditional flame retardants, so they are constantly the subject of much research to improve them [120,121].

The important feature of clays is their cation exchange capacity, which is quantitatively determining their ability to hold exchangeable cations. The important aspect of clays considering their flame-retardancy performance related to the cation exchange capacity is the possibility to modify them with various organic cations [122]. During such process, hydrated inorganic cations are replaced with organic ones. Considering almost unlimited number of potential organic cations, modification of clays may result in a very wide range of materials with various structural properties. From the flame-retardancy point of view, multiple organics may be applied to modify clays, e.g., alkylamine salts [123,124], phosphates [125,126], organic acids [127], chitosan derivatives [128], or other more complex compounds like 2-(2-(5,5-dimethyl-1,3,2-dioxaphosphinyl-2-ylamino)ethy-amino)-N,N,N-triethyl-2-oxoethanaminium chloride [129]. Such modifications enable introduction of nitrogen- and phosphorus-rich compounds onto the surface of clay particles, which, as mentioned above, is very beneficial for the flame-retardancy effects. Moreover, the use of compounds with bulky structure or with long hydrocarbon chain may lead to significant elongation of interlayer spacing and intercalation of clays, which noticeably facilitates their dispersion in polymer matrix [130].

## 4. Reduction of Polyurethane Foams’ Flammability by Clays

### 4.1. Impact on Thermal Decomposition Onset

**Figure 5:**
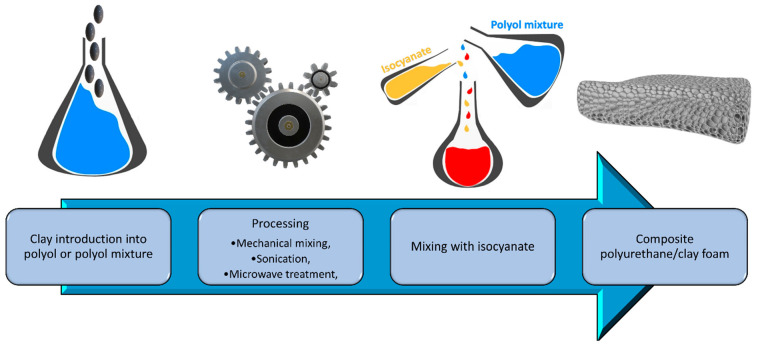
The conventional method for clay introduction into polyurethane foams.

Aluminosilicates are also quite popular for the modification of polyurethanes. These are naturally occurring compounds in the earth’s crust. Consequently, their availability and price are desirable, leading to the fact that they have been and continue to be the subject of much research over the years on improving PUR properties. Conventionally, clays are introduced into polyol mixture prior to foam preparation, as presented in Figure 5. As mentioned above, the introduction of nanometric aluminosilicate particles into the polymer improves its properties. What is mainly reported, the addition of aluminosilicate can improve the compressive and tensile strength, as well as Young’s modulus and elongation at break [131,132]. In addition to mechanical performance, other properties can be significantly improved, including the barrier properties important for thermal stability and flammability of material [133,134]. The addition of 4 wt% reduces the oxygen permeability by about 50% [135]. At the same time, the permeability of water and dichloromethane decreases with increasing the proportion of aluminosilicate to 20 wt% [136]. Such an effect is ascribed to the “labyrinth effect” caused by the presence of silicates nanoparticles (see Figure 4) [137,138]. Considering the flammability and thermal stability, it is essential because the heat and mass transfer of oxygen and degradation products in the composite is reduced. Therefore, the decomposition occurs slower, and the release of its products is not accelerating combustion as fast as in the unfilled polyurethane [25]. The literature reports on the enhancement of polyurethane foams’ thermal stability, as well as the values of char residue are summarized in Table 2.

**Figure 6:**
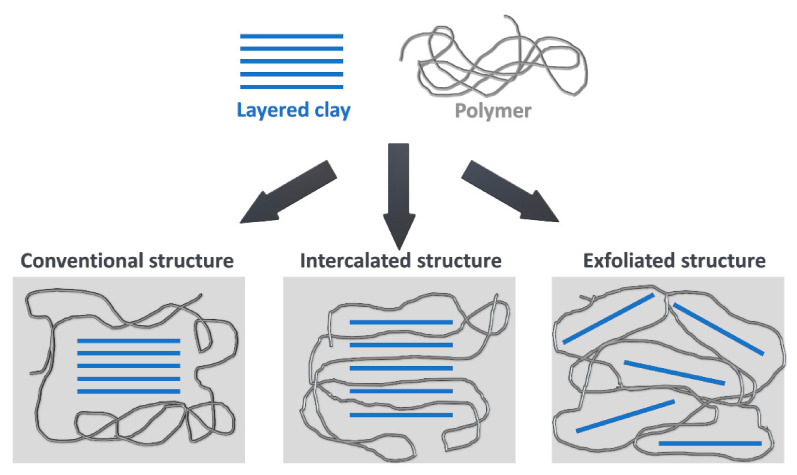
Different structure of clays in polymer matrix depending on the dispersion level.

It can be seen that in the case of rigid polyurethane foams, the nanoclays were found very efficient in enhancing thermal stability. Nevertheless, different effects were noted by various research groups, which could be associated with the level of filler dispersion in material and its potential intercalation or exfoliation [139]. Different levels of clay dispersion in polyurethane matrix are schematically presented in Figure 6. Its dispersion also influences the effectiveness of aluminosilicate modification in the composite, which can be significantly improved by sonication and other methods [140]. Heidarian et al. [141] studied the dispersion of clays in a polyol, which was later used to produce polyurethane by optical microscopy. Half-hour sonication significantly reduced the number and size of nanofiller agglomerates in the polyol compared to samples where only mechanical mixing was used. In terms of thermal stability, the enhanced dispersion of clays in polyurethane matrix results in a more effective heat barrier [142].

Considering the data presented for rigid polyurethane foams, the least significant effect of clay introduction was noted by Qi et al. [143]. They applied only the mechanical mixing of halloysite with polyol during the preparation of foams. Moreover, the shape of halloysite particles is usually more tubular compared to typical flake-shaped clays, which might significantly reduce the beneficial influence of potential “labyrinth effect”. As a result, the initial temperature of composites’ decomposition was increased only by 3–5 °C. On the contrary, Modesti et al. [144,145] introduced the microwave treatment to promote silicate dispersion in polyols. As a result, the intercalation of nanoparticles was noted by transmission electron microscopy and X-ray diffraction (XRD), and the onset of degradation was shifted by 13–17 °C towards higher temperatures. At the same time, a significant increase in the char residue content was noted, which could also be enhanced by the use of aluminum phosphinate as flame retardant along with the clay, pointing to the synergy effect. The simultaneous modification with flame retardant and nanoclay was found far more advantageous than using only one of these materials.

Considering the dispersion impact, Piszczyk et al. [146,147] noted more promising results, when additional sonication phase was introduced after mechanical mixing of clays with polyol mixture. Authors evaluated three types of clays as modifiers for rigid polyurethane foams bentonite, laponite, and montmorillonite, all of which were found very effective for thermal stability enhancement, increasing the onset of decomposition even by 39 °C for 6 wt% of laponite. Such a significant effect, noticeably higher compared to other reported works, was associated with the exfoliation of clay nanoparticles indicated by the XRD analysis.

**Figure 7:**
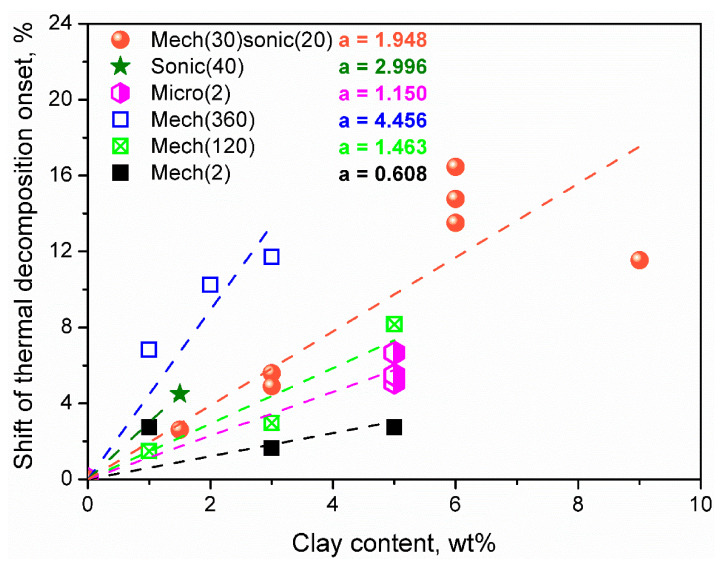
The impact of applied method of clay dispersion in polyol mixture on thermal stability enhancement (mech—mechanical mixing; micro—microwave treatment; sonic—sonication; and number in bracket indicate treatment time in min).

Figure 7 presents the impact of applied clay dispersion method prior to the foam preparation irrespectively of the type of foam, type of clay, its modifications, and presence of flame retardants. Nevertheless, despite these differences, it can be seen that elongation of the mechanical mixing, as well as introduction of microwave treatment and sonication, enhances thermal stability of PUR foams, which is associated with the improved clay dispersion, as indicated by the Authors. The most significant improvement of thermal stability, relatively to the initial temperature of decomposition of matrix, was noted by Panda et al. [151], who applied 360 min of mechanical mixing. As a result, exfoliated structure was obtained, as indicated by the XRD analysis.

Considering the relative effect, the 360 min mechanical mixing was more efficient than 30 min mechanical mixing combined with 20 min sonication, as proposed by Piszczyk et al. [146,147]. Based on the presented data, it can be assumed that the microwave treatment is the most effective method of clay dispersion in polyol mixture. The 2 min treatment applied by Modesti et al. [144] gave almost the same results as 120 min mechanical mixing proposed by Kausar [152]. At the same time, 1 min of sonication provided similar results to almost 6 min of mechanical mixing. Having in mind the above-mentioned differences in formulations of analyzed foams and types of applied clays, the efficiency of the dispersion methods decrease in the order microwave treatment > sonication > mechanical mixing.

For flexible foams the best results were observed by Kausar [150,153]. However, they were noted for poly(urethane-imide) and poly(urethane-ester) foams, which could provide additional possibilities for interactions with organoclay Cloisite 30B. The smallest increase in the initial decomposition temperature or even decrease was noted when clay was combined with organophosphorus flame retardants. Such an effect can be attributed to the decomposition of these materials and the generation of protective layers, which are supposed to enhance polymer materials’ fire resistance [157].

### 4.2. Impact on Limiting Oxygen Index

Generally, except for thermal stability and the onset of thermal decomposition, the beneficial effects of nanoclays, attributed to the “labyrinth effect” and reduced heat and mass transfer, were noted for the flammability of polyurethane foams. Even the tiny additions of nanoclays, up to 3 wt%, could reduce the oxygen permeability of polyurethane by more than 60% [135,158,159]. As a result, the modified PUR require the oxygen-richer atmosphere to burn, expressed by the higher values of their limiting oxygen index (LOI). This parameter indicates the minimum concentration of oxygen in the atmosphere, which supports the combustion of the material [160]. Table 3 summarizes the literature reports on the LOI value of foamed polyurethane/nanoclay composites.

Considering the flammability, LOI is one of the essential parameters of polyurethane materials. Compared to thermal stability, for LOI clays were more often examined in combination with other flame retardants, because researchers are looking for the synergy effects between them. Clays are often combined with the organophosphorus compounds, and the results are often quite promising [161,162]. The synergy can be well seen comparing the works of Danowska et al. [147] and Modesti et al. [144]. The initial values of LOI for unmodified polyurethane matrices were 22.5 and 20.6%, respectively. Application of 3 and 9% of Cloisite 30B caused the slight increase in LOI by 0.9 and 1.6%, respectively [147]. At the same time, the introduction of 10% of aluminum phosphinate along with the 5% of Cloisite 30B caused a significantly higher LOI increase up to 26.5% (5.9% rise) [144].

The synergy between clays and phosphorus-based flame retardants was also reported by Han et al. [164], who used diethyl bis(2-hydroxyethyl)aminomethylphosphonate to reduce the flammability of polyurethane foams. The incorporation of 2% of modified clay caused only a 0.4% rise in LOI. The effect was significantly more substantial after the addition of flame retardant, leading to an even 10.7% increase when 30% loading was used. Such an effect was also associated with the proper dispersion of clay in the matrix leading to the exfoliation of clay particles. As mentioned above, the proper dispersion of clay particles is essential and may significantly reduce the heat and mass transfer during combustion. Seo et al. [166] found that even a 15-min sonication of the samples enhanced the “labyrinth effect” of nanoclay by breaking up the filler agglomerates with ultrasound.

Summarizing the reports on the LOI changes, it can be seen that, compared to thermal decomposition onset, the presence of additional flame retardants is significantly more important.

### 4.3. Impact on the Results of Combustion Tests

**Figure 8:**
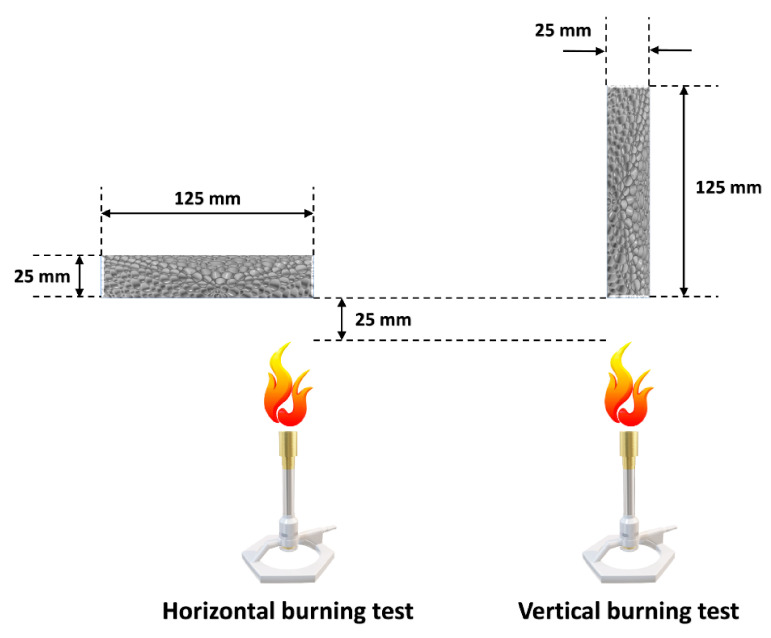
General scheme of horizontal and vertical UL94 burning tests.

The results presented in Table 3 indicate that the combinations of clays with conventional flame retardants may noticeably reduce the flammability of polyurethane foams, contrary to the use of clays alone, which do not guarantee non-combustibility. Literature data state that the addition of aluminosilicate nanofiller is often not enough for a sample to pass the horizontal or vertical combustion tests (UL 94, UL94HB, or ASTMD635), during which flame is applied directly to the specimen [167]. The general scheme of UL94 horizontal and vertical burning tests are presented in Figure 8.

Piszczyk et al. [156] performed the UL94 vertical burning test of flexible foams containing up to 9 wt% of montmorillonite/phosphorus flame-retardant mixture (50:50 mass ratio) or montmorillonite modified with phosphorus flame retardant. The flammability class could not be assigned because, irrespective of applied modifications, foams were burned entirely, or the required burning time was exceeded. However, the Authors indicated that the application of nanoclay reduced the dripping of material during combustion and slowed the combustion, which confirmed results presented by other researchers [134].

In the work of Danowska et al. [147], the introduction of 9 wt% of Cloisite 30B enabled reduction of combustion speed during horizontal burning from the initial 250 mm/min to 50 mm/min. The effect was noticeably stronger compared even to laponite, which was also exfoliated because of the presence of ternary ammonium salt. Nevertheless, it was not enough to obtain flammability class HB. A horizontal burning test was also performed by Wai et al. [168]. Unmodified polyurethane foam and its composites with modified montmorillonite (with tetraoctyl phosphonium bromide) and aluminum diethyl phosphinate were analyzed. Authors analyzed the combination of clay with flame retardant in the ratio of 100:0, 75:25, 50:50, and 25:75. The best results were noted for the 50:50 ratio, reducing the linear burning rate from 83.4 mm/min to 64.3 mm/min. When montmorillonite was applied without additional flame retardant, the burning rate was 72.6 mm/min. It points to the synergy between the applied modifiers and confirms previous works of other research groups, as well as the conclusions based on the LOI investigation [144]. Moreover, the 50:50 combination was also the most effective in reducing the heat of combustion determined by the bomb calorimeter.

### 4.4. Impact on the Cone Calorimetry Results

Except for the limiting oxygen index and above-mentioned burning tests, one of the most critical analyses associated with materials’ flammability is cone calorimetry. This method is crucial in the assessment of the fire behavior of materials. It enables the determination of multiple vital parameters characterizing the combustion process, such as heat release rate (HRR), total heat released (THR), total smoke released (TSR), a yield of generated gases, and others [157]. During the test, the material is subjected to heat irradiation, whose intensity should be in line with the particular fire situation, usually in the range of 25–75 kW/m^2^ [169]. Due to its importance, cone calorimetry is often applied during the assessment of polyurethane foams’ flammability. The literature reports on the cone calorimetry analysis of foamed polyurethane/clay composites are summarized in Table 4.

**Figure 9:**
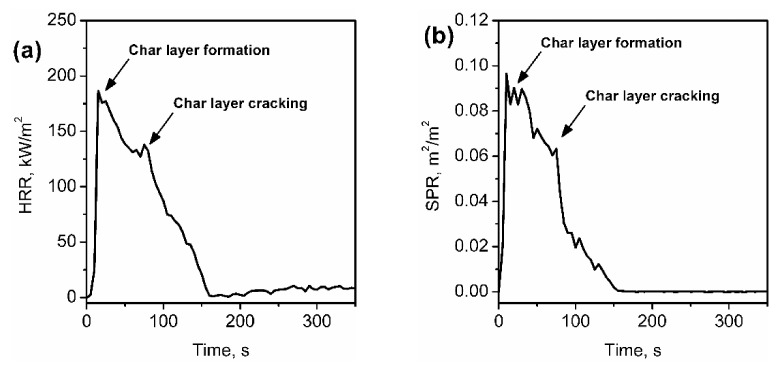
Typical HRR curve of polyurethane foam (**a**), and the effect of the char layer on smoke-production rate (SPR) (**b**).

One of the essential parameters of materials’ combustion determined by the cone calorimetry is the heat-release rate (HRR). It is directly associated with the rate of fire spread, hence the size of the fire, amount of generated heat, and gases. Higher values of the HRR implicates more difficulties in controlling the fire and more significant damage [26]. Therefore, the incorporation of flame retardants should efficiently reduce the HRR. Polyurethane foams themselves belong to the group of charring materials, for which the initial increase of HRR is noted during combustion until the efficient layer is formed, inhibiting the fire spread [170]. Table 4 indicates that in most of the reported cases, introduction of clays significantly reduced the peak value of the heat-release rate (pHRR), which is attributed to the formation of an additional char layer on the surface of the material [171]. It is related to the “labyrinth effect” and noticeably inhibited the release of combustion products from the material. As a result, HRR decreases over time, with the eventual momentary increase due to the cracking of the protective layer. Such an effect is very beneficial for fire safety and enables controlling of the fire. Figure 9 shows the typical HRR curve of polyurethane foams and the efficiency of the char layer on the reduction of smoke release from the material.

The incorporation of clays into foamed polyurethane matrix results in a very significant reduction of the peak HRR value, which indicates that compared to the unfilled matrix, the formation of the char layer is more efficient [172]. As a result, the fire is less intense and poses minor threats. Xu et al. [173] indicate that clays inhibit the combustion process, expressed by the decrease of the average mass loss rate of polyurethane composites. Such an effect is attributed to the reduced heat and mass transfer, mainly reducing the oxygen flow into the burning material. Despite the reduced intensity, the fire lasted longer, confirmed by other works [164,174]. Nevertheless, the impact of clay alone is often enough to reduce the total amount of heat released during combustion. It allows reducing the pHRR, which, as mentioned above, is a very beneficial effect but does not mean that clays always act like full-fledged flame retardants.

Similar to LOI, the clays were found the most beneficial when applied with other flame retardants. For solid polyurethanes, Tai et al. [175] reported a 64% reduction of pHRR value after introducing clay modified with polymeric flame retardant containing phosphorus and nitrogen atoms. In the case of foams, for the combination with organophosphorus compounds, the decrease in pHRR often exceeded 40% [163,164,176]. Such an effect was ascribed to the enhanced formation of a protective layer combined from the clay nanoparticles and phosphinates or phosphonates. Due to the “labyrinth effect,” more phosphorus was retained in the condensed phase, which was beneficial for flammability reduction. Moreover, organophosphorus flame retardants are considered very beneficial for gas-phase combustion due to the quenching effect of the free radicals generated during their decomposition [164].

The combustion in the gas phase is also described by the effective heat of combustion (EHC), which is the ratio of THR and mass loss rate. It corresponds with the degree of the burning of volatiles. Low values of the EHC are indicating that less heat is released from the volatile portion. Such an effect is very desirable for fire safety because it means less heat is transferred by the gas phase, and the spreading of the fire is limited [169]. The results presented by Han et al. [164] indicate that the incorporation of 2 wt% of clay reduced the EHC by 13%. The 6.2% reduction of TSR accompanies such an effect. When the clay was combined with diethyl bis(2-hydroxyethyl)aminomethylphosphonate, the enhancement of flame retardancy was noticeably higher. The effective heat of combustion was almost 20% lower, which could be associated with the above-mentioned gas-phase activity of organophosphorus flame retardant and a lower amount of smoke (16.5% reduction). It was attributed to the more efficient formation of the char layer, which was confirmed by the photographs of residual char and significantly higher values of char residue.

Such an effect was confirmed by the results presented by Zheng et al. [174] and Xu et al. [163]. The combination of clay with phosphorus flame retardants significantly reduced the smoke production rate due to the efficient formation of the char layer. The application of clay alone results in forming a very fragile char layer. The addition of organophosphorus compounds enables the formation of network structure, which results in the char layer densification, even though the X-ray diffraction analysis indicated no chemical reactions between clay and flame retardant [163]. Similar observations were made by Modesti et al. [144], who analyzed the char layer with a scanning electron microscope. Without clay, when only aluminum phosphinate was applied as flame retardant, the char layer showed a porous structure with noticeable holes, so it was not as effective in reducing heat and mass transfer. It was attributed to the partial vaporization of the phosphorus compound and its activity in the gas phase. Once again, it confirms the benefits of the joint application of clays and phosphorus flame retardants [179].

The results reported in the works mentioned above do not exclude the use of aluminosilicates for flame retardancy of polymer nanocomposites. Still, it shows that an appropriate flame retardant should be additionally used, which will allow obtaining synergism guaranteeing the flame retardancy of the product.

Interesting results on the flammability reduction of flexible polyurethane foams with hydrotalcite were also presented in the works of Gómez-Fernández et al. [154,155]. In the first work [155], the Authors applied unmodified clay and two variants intercalated with potassium phosphate monobasic and bis(2-ethylhexyl) hydrogen phosphate. In the second work [154], the same variants of hydrotalcite were introduced, but 5 or 10% of conventional polyol were replaced by the reactive type flame-retardant oligomer phosphonate—Exolit OP560. In both cases, fillers were introduced into polyurethane matrix in the amount of three parts per hundred of polyol. Unfortunately, formulations of prepared foams were not presented in the papers, so it is impossible to determine the actual content of clays in composites and compare the presented results in other works. Nevertheless, in Table 5, there are presented results obtained during pyrolysis combustion flow calorimetry analysis of prepared composites. It can be seen that for all formulations containing neat and modified clays and Exolit OP560, the flammability of the unfilled foam was reduced. The most promising results were reported when the organophosphorus partially replaced the conventional polyol. The combination of Exolit OP560 with unmodified hydrotalcite enabled an almost 21% reduction of peak heat-release rate. Quite effective was also modification of hydrotalcite with bis(2-ethylhexyl) hydrogen phosphate, contrary to the inorganic potassium phosphate monobasic. Such a phenomenon confirms the previous reports on the synergy between clays and organophosphorus flame retardants [161,162].

Summarizing, clays applied as fillers for polyurethane foams may effectively reduce their flammability. Such an effect is mostly related to the “labyrinth effect” caused by the small particle size and their flake-like shape, which significantly inhibits the heat, gas, and mass transfer during combustion. Nevertheless, as mentioned above, clays alone do not guarantee non-combustibility. Their efficiency can be noticeably enhanced by their modification or combining with conventional phosphorus-containing flame retardants, leading to the synergy effect. Moreover, the impact of clays can be significantly improved by the proper dispersion of filler, induced, e.g., by the microwave treatment or sonication.

## 5. Clay-Based Coatings for Polyurethane Foams

Except for the introduction of clays as a filler into a foamed polymer matrix, they can also be applied to reduce the flammability of polyurethanes via surface coating. It is an alternative approach, which involves the modification of previously prepared foam. The surface coating has been reported as an efficient method for flammability reduction [180]. Compared to the conventional incorporation of clays as fillers, surface coating shows a noticeably lower impact on the mechanical performance of foams, which is very beneficial. On the other side, such an approach requires additional operation after manufacturing foam and sometimes significantly increases the mass of foam [181]. Nevertheless, irrespective of the pros and cons compared to the above-described approach, the effectiveness of surface coating has been repeatedly proven [182,183].

**Figure 10:**
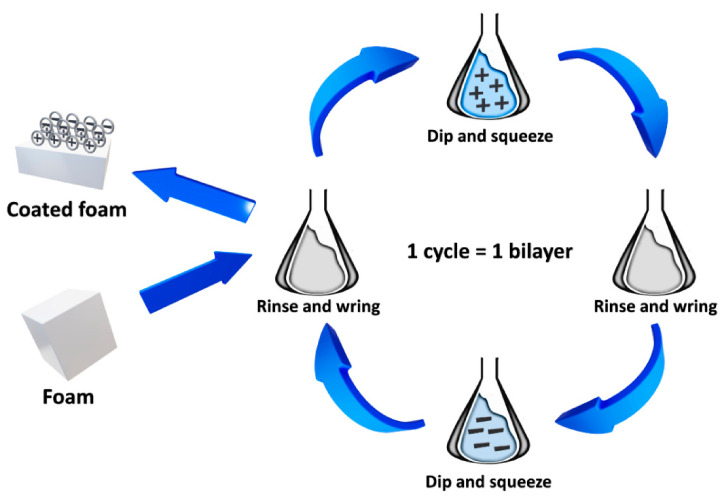
General scheme of layer-by-layer coating of polyurethane foams.

Clay-based coatings are deposited on the surface of polyurethane foams using the layer-by-layer assembly method (LbL). It is a relatively simple approach based mainly on the electrostatic interactions and enabling the preparation of coatings thinner than 1 μm [184]. During coating, oppositely charged solutions or suspensions are alternately deposited on coated material [181]. Each set of negative–positive pairs of compounds is called a bilayer. Except for the electrostatic interactions, the donor/acceptor interactions, hydrogen, or covalent bonding can be applied [185]. The general scheme of the procedure is presented in Figure 10.

Generally, LbL coatings can be divided into non-intumescent and intumescent, depending on their mechanism of action. The first group consists mainly of inorganic nanoparticles, which provide an inorganic ceramic barrier protecting the material during combustion. They noticeably reduce the heat, oxygen, and mass transfer limiting the damages caused by fire [186]. Non-intumescent organic coatings containing nanoparticles can be divided into zero-, one-, and two-dimensional, depending on the type of applied particles. Zero-dimensional coatings are based mainly on silica, aluminum, and their oxides [187]. One-dimensional contain various types of nanotubes, nanorods, or nanofibers [188]. Clays are widely applied in the preparation of two-dimensional coatings and other flake-shaped materials, like graphene oxide [189].

In most cases, clay-based coatings consist of anionic layers of poly(acrylic acid) (PAA) and cationic layers of polyethyleneimine (PEI). Clays are introduced into one of the layers or are entirely replacing them, usually anionic ones. Moreover, the clay layer may be introduced into an additional layer, resulting in trilayer coatings. Generally, the LbL assembly approach gives numerous possibilities for potential coatings, e.g., by adjusting polymers and clay concentrations or applying different amounts of bi- or trilayers [190].

**Figure 11:**
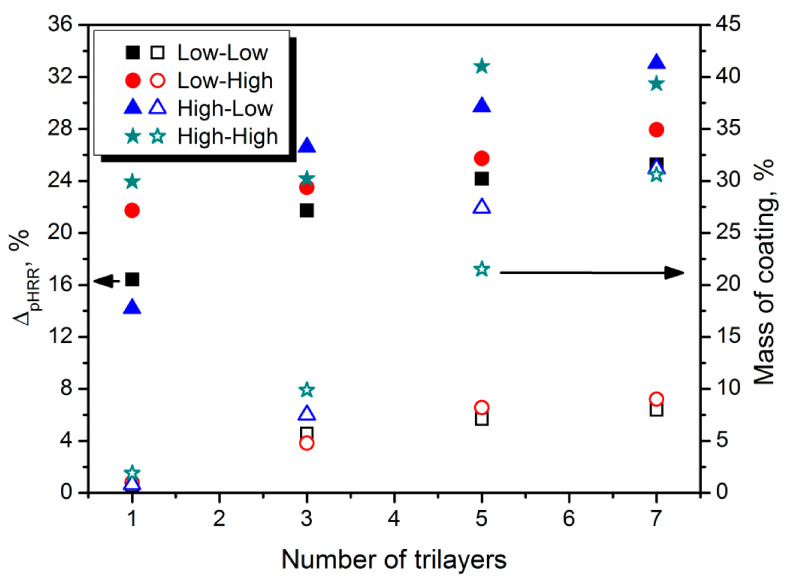
The impact of coating formulation on its mass and a resulting decrease of foam flammability [191].

Li et al. [191] prepared trilayer coating by introducing PAA anionic layer into PEI/clay coating. The impact of coating thickness (1, 3, 5, or 7 trilayers) and concentrations of particular components (0.1 or 0.5 wt% of polymers, 0.2 or 1.0 wt% of sodium montmorillonite) was evaluated. The impact of the applied coating formulation on its thickness and peak heat release rate was presented in Figure 11. Samples are coded according to the concentration of polymers and clay. All applied formulations were effectively enhancing the flame retardancy of the polyurethane foam, and for all of them, the efficiency was increasing with the coating thickness. However, the significant influence of clay on the efficiency was noted. Such an effect was associated with the higher quality of the char layer with hardly any imperfections, so the protective effect was more substantial. Nevertheless, except for the initial fire-protection effect, also the durability of the coating is very important, especially for the flexible materials. Therefore, polymer concentration should be maintained at the appropriate level to guarantee the flexibility of the coating. Comparing the low–high and high–high variants, higher content of polymer components led to higher durability and hardly any changes in flame retardant performance after compression cycles.

Li et al. [192] applied a similar procedure in their other work, but the sodium montmorillonite was replaced by layered double hydroxide (LDH). Trends in the coating mass and reduction of pHRR were very similar as for clay. However, quantitatively, the pHRR decrease was more significant, exceeding 42% for five trilayers of the high–high variant. Similar to MMT, the loading of layered double hydroxide showed an enormous impact on flame retardancy than polymer concentration. Increasing the loading of LDH in all cases led to the significant reduction of THR, exceeding even 25%. The effect for lower filler loading was noticeably weaker, and sometimes THR was even increased. It confirms the beneficial impact of filler introduction into LbL coatings on flammability reduction.

The results of these two works were combined by the Authors who evaluated the multilayer coatings containing both sodium montmorillonite and layered double hydroxide [193]. Different combinations comprising or bi-, tri-, and quadlayers were evaluated. Applied variants and their impact on the flammability of foams are summarized in Table 6. Presented results indicate that the LDH was more effectively enhancing flame retardancy of polyurethane foam. Independently on the applied coating sequence, the combination of LDH with MMT was more efficient than the use of MMT alone but less efficient than LDH alone. Such an effect confirms the results presented in previous works of the Authors [191,192]. Moreover, analysis of residue after combustion indicates that when LDH was introduced, significant amounts of polyurethane were detected in the post-test sample, confirming the higher efficiency of LDH compared to MMT.

Kim et al. [194] analyzed the incorporation of sodium montmorillonite into LbL PAA/PEI coatings. The clay (0.2 wt%) was introduced cationic, anionic, or both layers. Five bilayers were used to reduce the flammability of polyurethane foam. The introduction of clay into the PEI layer caused a significant drop of coating mass, compared to the sample with the PAA + MMT layer. When MMT was present in both phases, the mass of coating was the lowest. Nevertheless, in terms of flammability, the introduction of clay into both layers did not show any additional effects. For all variants, the pHRR was decreased by 25.1–27.3%. Moreover, the Authors investigated applying the prepared coating on the full-scale chair in the real-scale mockup test. Over 50% drop of pHRR was noted, and the size of the fire was significantly smaller. Such an effect was attributed to the additional impact of the covering fabrics, enhancing the coating impact. Moreover, coating resulted in a 13% increase in the residual mass of the chair.

Novel approaches to the preparation of LbL coatings for polyurethane foams include applying other, often more environmentally-friendly compounds to replace PAA or PEI. Among the most popular can be mentioned chitosan, starch, or alginates [195,196].

Cain et al. [197] coated polyurethane foam with chitosan/montmorillonite and chitosan/vermiculite (VMT) bilayers. However, the bilayer of PAA/PEI was applied to increase the adhesion between the foam and nanocoating. A significant reduction of flammability was noted even after the deposition of one clay layer on the PAA/PEI surface. The peak HRR was reduced by 27.8 and 53.9%, respectively, for montmorillonite and vermiculite. Also, other combustion parameters were reduced more significantly when VMT was introduced into coatings, which can be seen in Table 7.

The Authors attributed the advantages of vermiculite over montmorillonite to the higher aspect ratio of nanoparticles, which strengthened the “labyrinth effect” caused by the platelets and reduced the mass and heat transfer. Moreover, the energy-dispersive X-ray spectroscopy revealed that VMT clay contained the iron within tetrahedral layers, which might act as a radical trap and catalyze the charring, which also reduced the amount of generated smoke. The effectiveness of chitosan/vermiculite coating was later confirmed by Lazar et al. [198], who also applied the initial PAA/PEI bilayer to promote coating adhesion.

The high efficiency of vermiculite-based coatings was also confirmed by Holder et al. [199]. They compared the results of coating flexible polyurethane foam with chitosan/VMT and chitosan/ammonium polyphosphate (APP) bilayers. Similar results considering the pHRR decrease (around 55% drop) were noted for 4 layers of VMT and 20 layers of APP coatings. Stacked coating (first VMT, then APP) resulted in the 66% decrease of pHRR. Both coatings showed significantly different mechanisms of action. The APP-based layers noticeably reduced the amount of generated heat, but the TSR was more than doubled. On the other hand, the presence of vermiculite resulted in the formation of an efficient gas barrier, reducing the total amount of smoke by almost 57%. Presented work once again confirms that the combination of clays with phosphorus flame retardants may be auspicious for the flame retardancy of polyurethane materials.

Considering the application of chitosan, Laufer et al. [200] prepared a completely renewable LbL coating containing sodium montmorillonite without PAA or PEI. The impact of chitosan pH value was investigated (pH equal to 3 or 6). The chitosan particles were fully ionized at lower values, resulting in the self-repulsion of chains and the generation of very thin layers. When pH was increased to 6, conformations were more globular, thickening the layers and doubled the mass gain during coating. Moreover, it enabled better clay dispersion, which is very important for the performance of coating [201]. As a result, the coating created an almost complete gas barrier decreasing when 30 bilayers were assembled, which enhanced the flame retardancy effect. The pHRR was reduced by 36.9 and 52.4% for 10 bilayers, respectively, for pH 3 and 6. Presented results showed that fully green LbL coatings might be developed without the use of non-renewable polymers.

As mentioned above, except for the chitosan, another renewable and bio-based material that can be applied in LbL flame retardant coatings for polyurethanes is starch. Choi et al. [202] used it to prepare simple starch/MMT bilayer LbL coatings using spray-assisted assembly. The efficiency of the coating comprised of five bilayers was confirmed by a 22.7% drop of pHRR and a 52.7% reduction of THR comparing to the uncoated foam. It indicates that the char layer was efficiently formed during combustion. Moreover, prepared coating showed relatively good resistance to compression, which was evaluated by performance after 1000 compression cycles, according to the ASTM D3574 standard. Although the value of pHRR was slightly higher than for uncoated foam (2.7% increase), the value of total heat released was 57.8% lower. It points to the excellent adhesion between polyurethane foam and coating, which was not separated. The increase of pHRR points only to minor defects in the coating.

Zhang et al. [203] introduced potato starch (0.5 wt%) into the cationic layer during the preparation of montmorillonite or vermiculite (1.0 wt%) coatings comprised of 5 or 10 bilayers. Considering the cone calorimeter measurements, the results confirmed the above-mentioned reports of Cain et al. [197] about the high flame-retardancy effect of vermiculite. Even though the decrease of pHRR was very similar for MMT and VMT (52.9–54.0% and 55.2–58.5%, respectively, for 5 and 10 bilayers), the significant differences in THR and TSR parameters were noted. Due to the larger platelet size of VMT, the shielding was more efficient, and heat transfer was inhibited, so the THR values were ~4% lower than for the same amount of layers containing MMT. The reduction of gas transfer was even more substantial because VMT was around two times more efficient than MMT. The effectiveness of prepared coatings was confirmed by simple torch testing. Authors exposed foams to flame from the butane torch for 10 s. When vermiculite was applied as an anionic layer, the final residue exceeded 60%, even for 5 bilayers.

Davis et al. [204] presented a more complex procedure because, except for the montmorillonite, the combinations with sodium polyborate (SPB) were applied. Results obtained from the cone calorimeter and open flame tests are presented in Table 8. It can be seen that the combination of MMT and SPB provided excellent effects, considering not only the pHRR value but also significantly limiting the ignition of a sample, which can prevent the occurrence of fire at all. The coating containing 1.5 wt% of starch and 11.5 wt% of SPB was evaluated in a full-scale furniture test using a complete chair with fabrics. Depending on the fabric (thermoplastic or cotton), the pHRR was reduced by 63–75% compared to neat polyurethane foam. Moreover, during combustion, 55–71% less heat was released, which would be very beneficial during the fire and could significantly reduce the possibility of fire spread.

Considering the alginate use in LbL assembly coatings for flexible polyurethane foams, it was applied mostly by one research group. Pan et al. [205] applied it as a substitute for poly (acrylic acid) combined with PEI and sepiolite. The clay was introduced in the amount of 0.5 and 1.0 wt% into coating comprised of 3 or 6 bilayers. For comparison, coatings without clay were also prepared. The high efficiency of the coating process was confirmed by an energy-dispersive X-ray spectrometer, which indicated high contents of silicon and magnesium, the main elements present in sepiolite.

Table 9 presents the impact of applied coatings on the flame retardancy of polyurethane foam. In general, an unfilled coating comprising of PEI and alginate layers showed an excellent performance lowering the HRR peak by almost 40%. Moreover, despite the absence of clay nanoparticles causing the “labyrinth effect,” the amount of generated smoke and smoke production rate (SPR) was significantly reduced. The incorporation of sepiolite enhanced the flame-retardancy effect by the hindrance of heat transition during combustion. As a result, the pHRR was massively decreased by 76.1%, which is the most significant drop in heat-release rate among the reported literature data. Moreover, with the presence of clay, the char layer was noticeably stronger. As a result, the smoke was generated noticeably slower. For the highest content of sepiolite, the protective layer was primarily formed of clay particles, expressed by changes in char color. The residues maintained the initial shape of foam before the combustion, pointing to the high efficiency of the applied coating.

In their other work [206], the Authors also applied alginate as a PAA substitute in LbL coatings containing sodium montmorillonite and akaganeite (β-FeOOH) nanorods. Coatings containing only nanorods without MMT slightly improved the fire resistance of foam, but the effect was not very significant. The peak HRR was reduced by 14.1% for three trilayers. On the other hand, the application of MMT alone reduced pHRR by 44.6%, while the combination of nanoparticles by 46.6%. Such an effect was explained by the differences between char residues of particular samples. The network of nanorods was not strong enough to support the structure of foam during combustion and maintain the original shape of foam. As a result, the char residue collapsed utterly, which was confirmed by the SEM analysis. The montmorillonite alone provided more strength to the protective layer due to the enhanced charring, but the collapse was still observed. Incorporating both types of nanoparticles into alternating layers caused a synergy effect and provided sufficient support to the char layer that it retained well without collapse and fracture. Due to the high stability of the protective layer, the number of ethers, aromatics, carbonyl compounds, isocyanates, and hydrocarbons in smoke was significantly lowered, reducing the potentially toxic effects of smoke.

The renewable materials may also be combined since it has been proven that the chitosan/alginate coatings without the addition of flame retardants, clays, or other nanoparticles, may very effectively reduce the flammability of polyurethane foams [207].

Pan et al. [208] applied the combination of chitosan with alginate to prepare an LbL coating containing montmorillonite and carbon nanotubes. Clay was applied as a separate anionic layer, similar to alginate, while carbon nanotubes were introduced into the chitosan phase. Poly(acrylic acid) was applied as surface treatment of foam. The impact of clay loading (0.2 or 1.0 wt%) and a number of trilayers (4 or 8) was investigated. The increase in clay loading resulted in a more substantial fire inhibition effect. The pHRR was reduced by 43.1–60.1% and 65.9–69.4%, respectively, for 4 and 8 trilayers. A very significant reduction of heat release rate was ascribed to the presence of carbon nanotubes in the cationic layer, which acted as the “bridge” between clay nanoparticles and created a network limiting the heat and mass transfer during combustion. A similar effect was noted in work describing coatings containing MMT and β-FeOOH nanorods [206]. It indicates that among the nanoparticles, clays are among the essential ones in the preparation of fire protective LbL coatings for polyurethanes.

**Figure 12:**
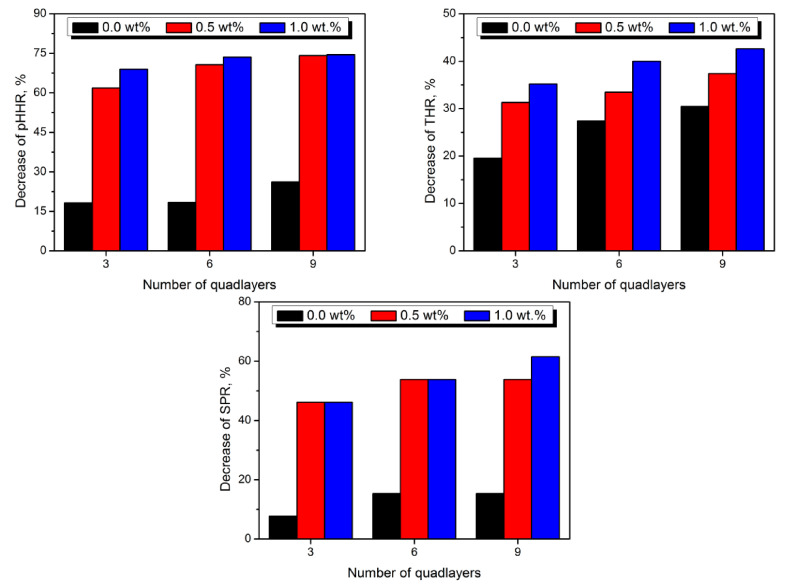
Polyurethane foams’ flammability reduction caused by the PEI/alginate/chitosan/laponite coating depending on the laponite content [196].

Recently, Nabipour et al. [196] prepared a quadlayer coating containing laponite and both chitosan and alginate. Nevertheless, the coating was not fully renewable because PAA was applied as an activator of the foam’s surface, and PEI was used as a positively charged layer. Without the addition of laponite, the coating already showed a positive impact on the foam’s flame retardancy, reducing the pHRR even by 26% and THR by 30%, which can be seen in Figure 12. However, the incorporation of laponite provided additional, very significant activity due to the increase in barrier effect. As a result, the smoke production rate was reduced four times more than for the PEI/chitosan/alginate coating. It was related to the lack of smoke-suppression effect of the organic layers. The Authors also reported essential information that the applied coating consisting of the nine quadlayers eliminated the melt dripping and induced self-extinguishing of polyurethane foam. According to provided information, such a combination was previously noted only by Holder et al. [199], who applied APP as one of the coating layers. Moreover, the Authors reported that the PEI/alginate/chitosan/laponite coating was the most effective in pHRR reduction (74% decrease) among the reported literature results. However, the above-mentioned work of Pan et al. [205] was not included in the analysis, probably due to the overlapping submission and acceptance dates of both articles.

Except for the work of Holder et al. [199], the APP was applied by Palen et al. [209], who used it to replace the PAA layer. The coating comprising its combination with PEI did not show any enhancement of flame retardancy. However, when the trilayer variant with the halloysite layer was applied, a 52.5% decrease in pHRR was observed due to synergy between phosphorus flame retardant and clay.

Generally, an excellent review work summarizing the advances in applications of layer-by-layer assembly technology in reducing polyurethane foams’ flammability was recently published by Liu et al. [190].

As mentioned above, the layer-by-layer assembly is by far the primary method applied for the coating of polyurethane foams. Hardly any works are reporting the use of other methods. Chen et al. [210] fabricated the alginate-based coatings by freeze-drying method. The prepared alginate/sodium montmorillonite suspensions were cast onto polyurethane foams, frozen in an ethanol/liquid nitrogen bath, and dried. The results were auspicious, attributed to the excellent flame-retardant performance of the prepared material. Without foam, a coating containing 7.5 wt% of both alginate and clays was characterized by the pHRR value of 20 kW/m^2^, THR of 4 MJ/m^2^, and TSR of 19.3 m^2^/m^2^. At the same time, unmodified foam showed values of 323 kW/m^2^, 20 MJ/m^2^, and 1527 m^2^/m^2^, respectively. The results of foam coating with prepared material are shown in Table 10.

To summarize, coating polyurethane foams aimed at reduction of their flammability looks pretty auspicious. It may often provide noticeably better results than the conventional approach, based on incorporating clays into the whole volume of the material. Such an approach shows multiple benefits but still requires a substantial amount of research.

## 6. Conclusions

The presented paper comprehensively summarized the literature reports related to reducing polyurethane foams’ flammability with clays, either by incorporating into the whole volume of material as a filler or by the coating of foam. The impact of various clays and their modification on the particular properties describing flammability of polyurethane foams was presented and tabulated. Considering thermal stability, the proper dispersion of clay and efficiency of “labyrinth effect” was found most essential. However, for fire resistance the proper clay modification or simultaneous incorporation of flame retardants was required, since clays alone, even exfoliated in matrix, were not able to inhibit combustion. Therefore, it is crucial to provide the synergy effect combining clays with flame retardants of modifying them. Possibly, clays could be applied as precursors for synthesis of flame-retarded polyols, which could be grafted onto the clay particles’ surface.

Coating polyurethane foams to reduce their flammability seems more advanced and a fascinating method and has already proven effective. Comparing to the conventional approach based on the introduction of clays as filler into the whole volume of foam, coating shows some pros and cons. First of all, it does not require the incorporation of clay into the foam during its preparation. Usually, fillers are mixed with polyols or the whole polyol mixture, where there are possibilities for potential interactions with other components, which may affect the system’s processing. The solid particles usually settle on the bottom of the container, implicating the need for additional mixing. Moreover, the presence of clay may noticeably affect the system’s viscosity and significantly impact its processing and the mechanical performance of the resulting foam. The coating-based approach is free of these disadvantages, and requires a lower amount of clay to provide similar fire protection, which can be economically beneficial, especially considering costly modification processes.

On the other hand, the coating requires additional operations after the actual manufacturing of foams, affecting the cost of production. The coating protects the surface of the foam, the protection in the whole volume is strictly depending on the share of open and closed cells in foam, which can limit the application of coatings in rigid foams. Moreover, even in flexible foams, the coating is required to show very high durability because of the damages that the external forces and deformation can cause, often present during the use of flexible foams. In many cases, the durability was hardly analyzed, so it is important to address this issue in further works. One can also argue about the actual industrial potential of LbL coatings, since the process is quite complex. Therefore, the efficiency of single bilayer coatings should be noticeably enhanced to simplify and shorten the whole modification process.

Reported data indicate that independently in the selected approach, the application of clays looks pretty auspicious. Nevertheless, it still requires much research to take full advantage of potential benefits. Future research works should focus on:

-  a better understanding of the synergy effects between clays and flame retardants, especially novel types of halogen-free, and possibly bio-based ones;
-  chemical modifications of clays, which would enhance their flame retardancy activity;
-  possible applications of clays as precursors for manufacturing of flame retardants and flame-retardant polyols;
-  modifications of clays, which, except for the flame retardancy, could provide other properties to foams, e.g., electrical conductivity;
-  the flammability of polyurethane/clay composites based on the novel, green raw materials;
-  the manufacturing of fully bio-based clay coatings without the use of additional synthetic compounds;
-  simplification of the coating process;
-  development of multi-purpose coatings, which could combine flame retardancy with other benefits for foams; and
-  evaluation of the novel techniques for coating polyurethane foams.

## Figures and Tables

**Table 1:** The most common combustion products of polyurethanes.

Combustion Product	Concentration, ppm	Effect on Humans after the Exposition	Ref.
Carbon oxide, CO	35	Headache and dizziness within 6 to 8 h of constant exposure	[64]
	100	Slight headache in 2 to 3 h	
	200	Slight headache within 2 to 3 h; loss of judgment	
	400	Frontal headache within 1 to 2 h	
	800	Dizziness, nausea, and convulsions within 45 min; insensible within 2 h	
	1600	Headache, tachycardia, dizziness, and nausea within 20 min; death in less than 2 h	
	3200	Headache, dizziness, and nausea in 5 to 10 min; death within 30 min	
	6400	Headache and dizziness in 1 to 2 min; convulsions, respiratory arrest, and death in less than 20 min	
	12,800	Unconsciousness after 2–3 breaths; death in less than 3 min	
Carbon dioxide, CO_2_	<40,000	Dilatation of cerebral vessels, increased pulmonary ventilation, and increased oxygen delivery to the tissues after 30 min	[65]
	40,000–70,000	Headache, hearing and visual disturbances, increased blood pressure, dyspnea, difficult breathing, depressions, and tremors	
	70,000–100,000	Unconsciousness, headache, visual and hearing dysfunction, depression, shortness of breath, and sweating after few minutes	
	100,000–150,000	Unconsciousness, severe muscle twitching, and dizziness after several minutes	
	>170,000	Loss of controlled activity, unconsciousness, convulsions, coma, and death within 1 min of initial inhalation	
Hydrogen cyanide, nitriles, R-CN	20–40	Headache, drowsiness, vertigo, weak and rapid pulse, rapid breathing, bright-red color in the face, nausea, and vomiting	[66]
	100	Fatal (1 h)	
	135	Fatal (30 min)	
	180	Fatal (10 min)	
	270	Rapidly fatal	
Nitrogen oxides, N_x_O_y_	25	Respiratory irritation, chest pain after 1 h	[67]
	50	Respiratory irritation, chest pain after 15 min; pulmonary edema possible subacute after 1 h	
	75	Pulmonary edema, possible subacute, and chronic lesions in the lungs after 30 min	
	100	Pulmonary edema, possible subacute, chronic lesions in the lungs after 15 min; death after 1 h	
	150	Pulmonary edema and death after 30 min	
	200	Possible subacute and chronic lesions in the lungs after 5 min; pulmonary edema, and death after 15 min	
	400	Pulmonary edema and death after 5 min	
Ammonia, NH_3_	50	Irritation to eyes, nose, and throat after 2 h	[68]
	100	Rapid eye and respiratory tract irritation	
	250	Tolerable by most persons (30–60 min exposure)	
	700	Immediately irritating to eyes and throat	
	>1500	Pulmonary edema, coughing, and laryngospasm	
	2500–4500	Fatal (30 min)	
	5000–10,000	Rapidly fatal due to airway obstruction	
Benzene, C_6_H_6_	500	Symptoms of illness after 1 h, slight irritation of mucous membranes	[69]
	1500	Serious symptoms	
	3000	Endurable	
	7500	Fatal (1 h), death associated with asphyxiation, respiratory arrest, and central nervous system depression	
	20,000	Fatal (5–10 min)	

**Table 2:** The impact of various clays and their modifications on the thermal stability of foamed polyurethane composites.

Foam Type	Clay	Modification of Clay	Flame Retardant	Clay Content, %	ΔT_ini_, °C	Δresidue, %	Ref.
Rigid foams	Bentonite	-	-	6	32	-	[146]
				9	7	-	[147]
	Cloisite 30B	methyl tallow bis-2-hydroxyethyl ammonium	-	3	16	-	[147]
				6	35	-	[146]
				9	33	-	[147]
			aluminium phosphinate, 10%	5	14	16.8	[144,145]
	Dellite HPS	-	aluminium phosphinate, 10%	5	13	17.0	
		[CH_3_OOCCH_2_(Ph)_2_PCH_2_CH_2_P(Ph)_2_CH_2_COOCH_3_]Br_2_		5	17	13.2	
	Halloysite	-	-	1	5	-	[143]
				3	3	-	
	Laponite RD	-	-	3	14	-	[147]
				6	39	-	[146]
	MMT K10	-	-	1	16	-	[148]
	Nanocor	PEG 600	-	2	25	1.0	[149]
		PEG 1000		2	7	3.0	
		PEG 1500		2	20	2.0	
Flexible foams	Cloisite 30B	methyl tallow bis-2-hydroxyethyl ammonium	-	1	44	15.0	[150]
				3	53	16.0	
				5	76	26.0	
				1	14	0.7	[151]
				2	21	1.2	
				3	24	2.0	
				1	6	-	[152]
				3	12	-	
				5	33	-	
				1	45	6.0	[153]
				3	49	8.0	
				5	55	11.0	
	Hydro- talcite	-	-	1.9	−1	1.6	[154,155]
		potassium phosphate monobasic		1.9	2	1.2	
			Exolit OP560, 5%	1.9	2	1.2	
			Exolit OP560, 10%	1.9	−7	2.7	
		bis(2-ethylhexyl) hydrogen phosphate	-	1.9	4	0.3	
			Exolit OP560, 5%	1.9	3	3.1	
			Exolit OP560, 10%	1.9	−8	3.1	
	Nanofil 116	-	Fyrol PNX, 1.5%	1.5	7	2.0	[156]
			Fyrol PNX, 3.0%	3	−19	5.0	
		Fyrol PNX	-	3	0	1.0	
				6	−1	3.0	

**Table 3:** The summary of the literature reports on the foamed polyurethane/clay composites’ limiting oxygen index.

Foam Type	Clay	Modification of Clay	Flame Retardant		Clay Content, %	ΔLOI, %	Ref.
Rigid foams	Cloisite 30B	methyl tallow bis-2-hydroxyethyl ammonium	-		3	0.9	[147]
					9	1.6	
			aluminium phosphinate, 10%		5	5.9	[144]
	Dellite HPS	-	aluminium phosphinate, 10%		5	5.1	
		[CH_3_OOCCH_2_(Ph)_2_PCH_2_CH_2_P (Ph)_2_CH_2_COOCH_3_]Br_2_	aluminium phosphinate, 10%		5	6.1	
	MMT	octadecyl bis-hydroxyethyl methyl ammonium chloride	ammonium phosphate, 8%		5	6.5	[163]
			dimethyl methyl phosphonate, 8%		5	7.5	
			ammonium phosphate, 8% + dimethyl methyl phosphonate, 8%		5	9.5	
	Na MMT	hexadecyltrimethyl ammonium bromide	-		2	0.4	[164]
			Diethyl bis(2-hydroxyethyl)aminomethylphosphonate	10%	2	4.1	
				20%	2	8.4	
				30%	2	10.7	
	Nanofil 2	-	-		1.2	1.7	[165]
			zinc stannate, 1.2%		1.2	2.3	
			zinc stannate, 1.1% + expandable graphite, 2.2%		1.1	3.4	
			zinc stannate, 1.1% + expandable graphite, 6.5%		1.1	5.0	
Flexible foams	Cloisite 30B	methyl tallow bis-2-hydroxyethyl ammonium	-		1	2	[152]
					3	7	
					5	9	
					1	5	[153]
					3	7	
					5	10	

**Table 4:** Summary of the literature reports on the polyurethane/clay composites flammability reduction determined by cone calorimetry.

Clay	Modification of Clay	Flame Retardant	Clay Content, %	Δ_pHRR_, %	Δ_THR_, %	Δ_TSR_, %	Δ_char_, %	Ref.
Cloisite 30B	methyl tallow bis-2-hydroxyethyl ammonium	aluminum tris-diethylphosphinate, 10%	3	40.9	30.3	-	-	[176]
			5	35.5	27.3	-	-	
		aluminum tris-diethylphosphinate + melamine cyanurate, 10%	3	40.2	33.3	-	-	
			5	44.4	27.3	-	-	
		aluminium phosphinate, 10%	5	27.2	12.9	4.8	10.7	[144]
Dellite HPS	-	aluminium phosphinate, 10%	5	22.1	14.2	10.5	8.7	
	[CH_3_OOCCH_2_(Ph)_2_PCH_2_CH_2_P(Ph)_2_CH_2_COOCH_3_]Br_2_		5	35.9	18.2	4.3	5.9	
MMT	-	-	5	0.0	3.3	-	18.7	[177,178]
			10	3.0	11.5	-	26.9	
			15	0.0	24.4	-	31.0	
		ammonium polyphosphate + pentaerythritol (2:1 ratio), 5%	5	5.1	9.2	-	15.8	
		ammonium polyphosphate + pentaerythritol (2:1 ratio), 10%	5	17.9	29.7	-	23.8	
	octadecyl bis-hydroxyethyl methyl ammonium chloride	ammonium phosphate, 8% + dimethyl methyl phosphonate, 8%	5	42.5	-	-	-	[163]
		-	5	12.4	-	1.0	-	[174]
		ammonium polyphosphate, 8% + triphenyl phosphate, 4%	5	33.7	-	28.0	-	
	octadecyl trimethyl ammonium	-	0.8	15.9	-	-	-	[173]
Na MMT	octadecyl primary ammonium	-	0.8	30.2	-	-	-	
	decanediamine		0.8	26.4	-	-	-	
	hexadecyltrimethyl ammonium bromide		2	19.1	6.2	5.7	6.3	[164]
		Diethyl bis(2-hydroxyethyl)aminomethylphosphonate, 30%	2	50.5	43.8	16.5	20.3	

**Table 5:** The results of the pyrolysis combustion flow calorimetry analysis of polyurethane/hydrotalcite composite foams [154,155].

Hydrotalcite Modification	Content of Exolit OP560 in Polyol Mixture, %	pHRR, W/g	THR, kJ/g
Reference foam (no hydrotalcite)	0	144.7 ± 6.5	28.2 ± 0.6
-	0	136.4 ± 5.5	26.9 ± 0.4
	5	132.7 ± 6.5	26.1 ± 0.5
	10	114.7 ± 7.3	25.9 ± 0.2
potassium phosphate monobasic	0	138.4 ± 6.5	26.5 ± 0.5
	5	136.0 ± 5.5	26.2 ± 0.2
	10	138.8 ± 6.2	25.6 ± 0.5
bis(2-ethylhexyl) hydrogen phosphate	0	133.2 ± 11.0	27.4 ± 0.8
	5	121.4 ± 6.0	26.2 ± 0.5
	10	131.0 ± 7.4	25.9 ± 0.3

**Table 6:** The influence of applied coatings on the fire performance of flexible polyurethane foams [193].

Type of Coating	Coating Sequence	Content				Coating Mass, %	Nanoparticle Content, %	Δ_pHRR_, %	Δ_THR_, %
		PAA	PEI	LDH	MMT				
Bilayer	PAA/PEI + LDH	0.2	0.2	0.5	-	25.0	54	40	14
	PAA/PEI + LDH			1.0	-	30.0	58	39	19
	PAA + MMT/PEI			-	0.5	11.0	32	22	0
	PAA + MMT/PEI + LDH			0.5	0.5	11.0	32	27	7
Trilayer	PAA/PEI + LDH/MMT			0.5	0.5	11.0	43	28	9
	PAA/PEI + LDH/MMT			1.0	1.0	7.5	64	29	16
Quadlayer	PAA/LDH/PEI/MMT			0.5	0.5	13.0	29	33	5
	PAA/LDH/PEI/MMT			1.0	1.0	8.4	56	31	21

**Table 7:** The effects of MMT and VMT coatings on the flammability of polyurethane foams [197].

Coating and Number of Bilayers	Δ_pHRR_, %	Δ_THR_, %	Δ_residue_, %	Δ_TSR_, %
MMT 1	27.8	4.1	6.0	−7.5
MMT 2	53.3	1.5	4.0	11.0
MMT 4	59.5	11.8	8.0	50.7
VMT 1	53.9	8.2	13.0	30.8
VMT 2	56.2	12.3	11.0	58.2

**Table 8:** The impact of the coating composition on the peak heat release rate and open-flame test results of polyurethane foams [204].

Starch Content, wt%	SPB Content, wt%	MMT Content, wt%	Δ_pHRR_, %	Open-Flame Test Results
1.5	5.8	-	42	Self-extinguished < 40 s
	5.8	2.0	60	Self-extinguished < 20 s
	11.5	-	64	Self-extinguished < 20 s
	11.5	2.0	66	No ignition
3.0	5.8	-	53	Self-extinguished < 20 s
	5.8	2.0	63	Self-extinguished < 20 s
	11.5	-	66	No ignition
	11.5	2.0	66	No ignition
	23.0	-	75	No ignition

**Table 9:** Flammability reduction of polyurethane foams caused by the sepiolite-based coatings [205].

Number of Bilayers	Sepiolite Content, wt%	Δ_pHRR_, %	Δ_THR_, %	Δ_SPR_, %	Δ_TSR_, %
3	0.0	25.4	4.9	33.3	9.8
6		39.4	12.9	41.7	19.5
3	0.5	60.6	14.4	58.3	22.0
6		70.4	19.3	58.3	29.3
3	1.0	76.1	24.2	58.3	24.4
6		76.1	23.9	58.3	26.8

**Table 10:** The influence of freeze-dried alginate-clay coatings on the flammability of polyurethane foams [210].

Alginate (A) and Clay (C) Content, wt%	Coating Thickness, mm	Δ_pHRR_, %	Δ_THR_, %	Δ_TSR_, %	Δ_residue_, %
A5C5	1.5	55.4	−5	39.2	14.7
A5C10	1.5	65.9	20	62.0	43.6
A7.5C7.5	0.2	31.0	5	46.5	9.3
	0.7	31.9	5	36.9	19.6
	1.5	60.4	5	46.3	33.9

## Data Availability

Data are contained within the article. The data presented in this study are available in Clays as inhibitors of polyurethane foams’ flammability.

## Abbreviations

APP	ammonium polyphosphate
ATH	aluminum hydroxide
CASE	coatings, adhesives, sealants, and elastomers
EHC	effective heat of combustion
HRR	heat-release rate
LbL	layer-by-layer assembly
LDH	layered double hydroxide
LOI	limiting oxygen index
MDH	magnesium hydroxide
MMT	montmorillonite
PA	polyamide
PAA	poly(acrylic acid)
PEI	polyethyleneimine
PET	poly(ethylene terephthalate)
pHRR	peak value of heat-release rate
PP	polypropylene
PUR	polyurethane
PVC	poly(vinyl chloride)
SPB	sodium polyborate
SPR	smoke production rate
TDI	*p*-toluyleneisocyanate
THR	total heat released
TSR	total smoke released
VMT	vermiculite
XRD	X-ray diffraction
